# Cytokinin cross-talking during biotic and abiotic stress responses

**DOI:** 10.3389/fpls.2013.00451

**Published:** 2013-11-19

**Authors:** José A. O’Brien, Eva Benková

**Affiliations:** ^1^Department of Plant Systems Biology, VIB, GentBelgium; ^2^Department of Plant Biotechnology and Bioinformatics, Ghent University GentBelgium; ^3^Institute of Science and Technology AustriaKlosterneuburg, Austria

**Keywords:** cytokinin, stress, hormonal crosstalk, salicylic acid, abscisic acid

## Abstract

As sessile organisms, plants have to be able to adapt to a continuously changing environment. Plants that perceive some of these changes as stress signals activate signaling pathways to modulate their development and to enable them to survive. The complex responses to environmental cues are to a large extent mediated by plant hormones that together orchestrate the final plant response. The phytohormone cytokinin is involved in many plant developmental processes. Recently, it has been established that cytokinin plays an important role in stress responses, but does not act alone. Indeed, the hormonal control of plant development and stress adaptation is the outcome of a complex network of multiple synergistic and antagonistic interactions between various hormones. Here, we review the recent findings on the cytokinin function as part of this hormonal network. We focus on the importance of the crosstalk between cytokinin and other hormones, such as abscisic acid, jasmonate, salicylic acid, ethylene, and auxin in the modulation of plant development and stress adaptation. Finally, the impact of the current research in the biotechnological industry will be discussed.

## INTRODUCTION

During their lifespan, plants are exposed to continuously changing environmental conditions and pathogen threats. Various abiotic and biotic stresses, such as heat, cold, drought, high salinity, or pathogen attacks, can severely affect plant development, growth, fertility, and productivity. To survive, plants must be able to react rapidly to various stress signals, activate efficient defense responses, and adapt to new conditions. Plant hormones are key components of these defense and adaptation mechanisms. To mediate the responses and adaptations to stresses, different hormonal pathways are upregulated or downregulated. Modifications in the hormonal abundance and signaling will usually impact on the degree of resistance or susceptibility to the various stresses.

## HORMONES AND ABIOTIC STRESSES

Plants can perceive and respond to environmental changes. For instance, seasonal variations in day/night length or in temperature might directly affect the reproductive cycle, flowering, and fruit set. However, unpredicted changes, such as flooding, extreme temperature, heavy metals, drought, or high salt levels, will be perceived as stress conditions and might have a strongly negative impact on grain yield, grain weight, and plant biomass. Likewise, the root system architecture will adapt in terms of growth and branching as a reaction to different stresses. Among the various stress conditions, salinity and drought are currently the major problems. Saline soils represent a total of 323 million hectares worldwide (Brinkman, 1980), whereas drought affects 1–3% of the land surface and is predicted to increase to up to 30% by 2090 (Burke et al., 2006). To cope with these stresses, plants modify the levels of the different phytohormones directly or indirectly. This altered hormonal balance also affects the plant development, with a direct impact on seed development, seed germination, dormancy, and overall plant growth (Finkelstein et al., 2002).

### ABSCISIC ACID –THE ABIOTIC STRESS HORMONE

In response to abiotic stresses, such as drought and salinity, endogenous abscisic acid (ABA) levels increase rapidly, activating specific signaling pathways and modifying gene expression levels (Seki et al., 2002; Rabbani et al., 2003; Kilian et al., 2007; Goda et al., 2008; Zeller et al., 2009). In fact, up to 10% of protein-encoding genes are transcriptionally regulated by ABA (Nemhauser et al., 2006).

Abscisic acid is one of the most studied phytohormone because of its rapid response and prominent role in plant adaptation to abiotic stresses. In the meantime, the key components of the ABA signaling pathway have been characterized (Sreenivasulu et al., 2007; Cutler et al., 2010; Hirayama and Shinozaki, 2010; Raghavendra et al., 2010; Debnath et al., 2011; Fujita et al., 2011). In *Arabidopsis thaliana*, the pyrabactin resistance1 (PYR1)/PYR1-LIKE (PYL)/regulatory components of ABA receptor (RCAR) proteins have been proposed as the main intracellular ABA receptors (Ma et al., 2009; Park et al., 2009; Santiago et al., 2009a; Nishimura et al., 2010). Multiple ABA receptor loss-of-function mutants, such as *pyr1/pyl1/pyl4*, *pyr1/pyl1/pyl2/pyl4,* and *pyr1/pyl1/pyl2/pyl4/pyl5/pyl8 *are insensitive to ABA, even at concentrations as high as 100 μM (Park et al., 2009; Gonzalez-Guzman et al., 2012). Particularly, the quadruple and sextuple mutants were less sensitive to the ABA-mediated inhibition of seed germination, root growth, stomata closure, and expression of ABA responsive genes (Park et al., 2009; Nishimura et al., 2010; Gonzalez-Guzman et al., 2012). Accordingly, *PYL5* overexpression resulted in high drought resistance and an enhanced response to ABA (Santiago et al., 2009b).

In the presence of ABA, the PYR/PYL/RCAR proteins form a ternary complex that via direct interaction inhibit clade A protein phosphatase 2C (PP2C), including ABA-INSENSITIVE 1 (ABI1), ABI2, and hypersensitive to ABA 1 (HAB1) (Nishimura et al., 2007; Santiago et al., 2009a; Szostkiewicz et al., 2010). Similarly to the receptor mutants, mutants in the PP2C activity, such as *abi1-1*, are also insensitive to ABA (Fujii and Zhu, 2009; Cutler et al., 2010). PP2C repression activates downstream targets, such as the protein kinases belonging to the sucrose non-fermenting 1-related subfamily2 SnRK2.2/D, SnRK2.3/I, and SnRK2.6/OST1/E, which trigger ABA-dependent gene expression and signaling (Umezawa et al., 2009; Vlad et al., 2009). Accordingly, the *snrk2.2*/*snrk2.3*/*snrk2.6* triple mutant is highly insensitive to ABA and severely affects plant growth and seed yield (Fujii and Zhu, 2009).

### CYTOKININ IN ABIOTIC STRESS RESPONSES

Besides ABA, other hormonal pathways, including cytokinin (CK), are activated when a plant is exposed to stress. The CK-dependent modulation of stress responses has been studied at various levels. The alteration of endogenous CK levels in reaction to stress suggests that this hormone is involved in stress responses. For instance, in response to drought, the *in planta* concentration and transport of *trans*-zeatin riboside decreases drastically, whereas the ABA levels increase (Hansen and Dörffling, 2003; Davies et al., 2005). Interestingly, when the partial root zone-drying approach was applied, the CK concentration decreased, not only in roots, but also in leaves, buds, and shoot tips, along with increased ABA levels (Stoll et al., 2000; Kudoyarova et al., 2007). These observations demonstrate that the local stress exerted on the root might trigger changes in the CK levels in various plant organs, including the shoot, and, consequently, in developmental processes, such as the apical dominance (Hansen and Dörffling, 2003; Schachtman and Goodger, 2008). Typically, reduced CK levels would enhance the apical dominance, which, together with the ABA regulation of the stomatal aperture, aids to adapt to drought stress.

The negative CK-regulatory function in plants exposed to drought has been demonstrated in genetic studies in which the endogenous CK levels were modified, either by loss of the biosynthesis genes isopentyl transferase (IPT) or by overexpression of cytokinin oxidase (CKX)-encoding degradation genes (Werner et al., 2010; Nishiyama et al., 2011; Wang et al., 2011b). A reduced CK content in the *ipt1/ipt3/ipt5/ipt7* quadruple and *ipt8* single mutants or overexpression of *CKX1 *and its homologs**correlates with an increased resistance to both salt and drought stresses.

In agreement with the increased abiotic stress resistance at low CK levels, mutants lacking the functional CK receptors are more resistant to abiotic stresses (Tran et al., 2007; Jeon et al., 2010; Kang et al., 2012). For example, the *Arabidopsis histidine kinase* (*AHK*) loss-of-function mutants *ahk2/ahk3* and *ahk3/ahk4* were significantly more resistant to freezing temperatures than the wild type (Jeon et al., 2010). Similarly, all *ahk* single and multiple mutants, with the exception of *ahk4*, showed an enhanced resistance to dehydration (Kang et al., 2012). Furthermore, like the CK-metabolic mutants *ipt1/ipt3/ipt5/ipt7*, *ipt8,* and the *CKX1*-overexpressing plants, the *ahk* mutants affected dramatically the ABA sensitivity (Tran et al., 2007) and were hypersensitive to ABA treatments.

**FIGURE 1:**
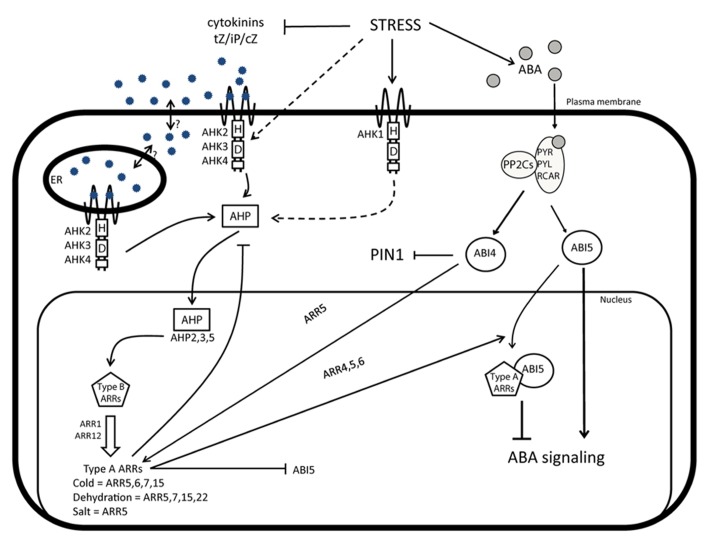
**CK and crosstalks during abiotic stress responses.** Under non-stress conditions, CK activates signaling mediated through AHK receptors, AHPs, and type-B response regulators ARRs. Type-B ARRs stimulate the expression of the early CK response genes, including type-A *ARR* genes that provide a negative feedback loop of the CK signaling. Besides this negative feedback loop, type-A ARRs also repress the expression of *ABI5* and interfere with the ABA signaling, through the physical interaction with ABI5. In response to stress, ABA levels increase and, simultaneously, CK levels decrease. The recognition of ABA by the receptors PYR/PYL/RCAR promotes the interaction with PP2C proteins that will activate downstream responses through signaling components including ABI5 and ABI4. At the same time, ABA interferes with the activity of CK and auxin and via ABI4 attenuates the expression of the *PIN1* auxin efflux carrier and enhances the transcription of the CK signaling repressor *ARR5*. Interestingly, type-A ARRs, such as ARR5, are upregulated, despite the low CK levels, probably because of the indirect activation of the CK signaling pathway by alternative receptors of the histidine kinase family, such as AHK1.

Downstream of the AHK receptors, the *Arabidopsis* histidine phosphotransfer (AHP) proteins mediate stress signaling (Hwang and Sheen, 2001; Hutchison et al., 2006; To and Kieber, 2008; Hwang et al., 2012). AHP proteins translocate into the nucleus and activate the type-B *Arabidopsis* response regulator (ARR) factors that trigger the transcription of specific genes in response to CK. A negative feedback loop is provided by type-A ARRs that inhibit the activity of type-B ARRs by a still unknown mechanism (**Figure 1**). Of all ARRs, type-A ARRs are the only ones of which the expression is altered under stress, e.g., *ARR5*, *ARR6*, *ARR7*, and *ARR15* are upregulated upon cold stress (Jeon et al., 2010; Jeon and Kim, 2013); *ARR5*, *ARR7*, *ARR15,* and type-C *ARR22* are upregulated in response to dehydration (Kang et al., 2012); and *ARR5* expression increases in response to salt stress (Mason et al., 2010). Stimulation of *ARR5*, *ARR6*, *ARR7,* and *ARR15* expression in response to cold stress requires the activity of several components of the CK signaling pathway, including AHP2, AHP3, and AHP5, and also ARR1 (Jeon and Kim, 2013). Likewise, in response to salt stress, *ARR5* upregulation depends on *ARR1* and *ARR12* (Mason et al., 2010). Furthermore, the negative regulatory role of AHP2, AHP3, and AHP5 during drought stress has been described recently (Nishiyama et al., 2013).

Despite the clear indications that CK and the CK signaling components function in stress responses (Hwang et al., 2012), the high degree of redundancy in the CK signaling pathway, including three CK receptors, six AHPs, 10 type-A ARRs, and 11 type-B ARRs, makes it difficult to dissect the role of each specific component (Hwang et al., 2012). Interestingly, although CK levels are reduced, the type-A ARRs that belong to the early CK-responsive genes are upregulated (Jeon et al., 2010; Mason et al., 2010; Kang et al., 2012; Jeon and Kim, 2013). Furthermore, a quadruple type-A ARR loss-of-function mutant *arr3/arr4/arr5/arr6* is resistant to salt stress, which is unexpected because to type-A ARRs act as CK signaling repressors (Mason et al., 2010). These observations imply that in stress responses the role played by the CK signaling pathway is more complex. In this context, AHKs might function as stress sensors that would activate the CK signaling pathway independently of CK levels (Urao et al., 1999; Tran et al., 2007; Jeon et al., 2010). In fact, another member of the histidine kinase family, AHK1, is able to sense and transduce changes in osmolarity to trigger downstream signaling pathways (Urao et al., 1999; Tran et al., 2007). However, unlike the CK receptors AHK2, AHK3, and AHK4, AHK1 positively regulates stress responses. Thus, it remains to be elucidated whether AHK2, AHK3, or AHK4 can sense abiotic stresses independently of CK, or whether AHK1 might crosstalk with a downstream CK signaling cascade.

Besides core components of the CK transduction cascade, downstream targets in stress responses have been disclosed as well. The cytokinin response factor (CRF) transcription factors of the APETALA2 (AP2) family have been identified as early CK response genes of which the expression is rapidly induced after CK application (Rashotte et al., 2006). Interestingly, the *CRF6* homolog is also highly responsive to various abiotic stress treatments (Zwack et al., 2013) and, recently, its regulatory role has been characterized in leaf senescence control (Zwack et al., 2013).

### HORMONAL CROSSTALKS AND ABIOTIC STRESS RESPONSES

The altered ABA sensitivity in plants with modified CK levels and signaling (Tran et al., 2007; Werner et al., 2010; Nishiyama et al., 2011; Wang et al., 2011b) hints at a crosstalk between ABA and CK. Interestingly, ARR4, ARR5, and ARR6 have been found to interact with ABI5 and also to regulate its expression levels. ABI5 is a basic leucine zipper protein that positively regulates the ABA signaling. The interaction with type-A ARRs attenuates the ABI5 activity and suppresses the ABA signaling (**Figure 1**; Wang et al., 2011b). Thus, type-A ARRs might, in addition to their regulation of the CK signaling, also control ABA signaling.

New insights into the ABA-CK crosstalk have been gained from the functional analysis of ABI4 (Shkolnik-Inbar and Bar-Zvi, 2010), that belong to the AP2 family of transcription factors. Similar to ABI5, ABI4 is also a positive regulator of the ABA signaling (Wind et al., 2013) and of the type-A *ARR5* expression that represses the CK signaling. Simultaneously, ABI4 attenuates the expression of the *PIN-FORMED 1* (*PIN1*) gene, an auxin efflux carrier that is an essential component of the polar auxin transport machinery (Shkolnik-Inbar and Bar-Zvi, 2010). Thus, ABI4 might represent an important crosstalk point on the interface of ABA, CK, and auxin pathways (**Figure 1**), in agreement with observations demonstrating that both the levels of CK and auxin, as well as of the PIN3 and PIN7 auxin efflux carriers, are suppressed when the ABA level increases (Hwang and Sheen, 2001; Wang et al., 2011a). Altogether, the strong impact of stress on plant development might result from the combined activities of several hormonal pathways, such as ABA and development-related hormones, such as CK and auxin.

The hormonal pathway of ethylene (ET) contributes also to the complexity of the hormonal network underlying plant responses to stresses. ET has been studied both in a developmental and stress context (Cary et al., 1995; Chae et al., 2003; Dietz et al., 2010; Kushwah et al., 2011; Beguerisse-Díaz et al., 2012; Vanstraelen and Benková, 2012; Zhai et al., 2013) and, recently, its role as a negative regulator of freezing tolerance has been demonstrated (Shi et al., 2012). The ET activity in stress responses is mediated by the downstream transcription factor of the ET signaling cascade, ethylene-insensitive 3 (EIN3). EIN3 suppresses the expression of the *C-repeat/dehydration response element-binding factor 1* (*CBF1*), *CBF2,* and *CBF3* genes, which mediate the response to cold stress, and also of the CK signaling repressors *ARR5*, *ARR7,* and *ARR15* by direct binding to their promoters (Shi et al., 2012). Although ET interferes with the CK signaling output, its pathway is also affected by CK. Indeed, CK stabilizes 1-aminocyclopropane-1-carboxylate synthase 5 (ACS5) and ACS9 (Vogel et al., 1998; Chae et al., 2003; Hansen et al., 2009) that convert *S*-adenosyl-methionine to 1-aminocyclopropane-1-carboxylic acid (ACC), the rate-limiting step in the ET biosynthesis. This stabilization might lead to an ET accumulation and, consequently, affect plant growth processes, such as root growth (Cary et al., 1995;  Růžička et al., 2007). The complexity of the hormonal regulatory network underlying stress responses has been suggested (Lehotai et al., 2012) by the activation of both CK and ET signaling in response to selenite-induced stress by means of the ARR5 and ACS8 markers and decrease in the auxin levels.

Interestingly, the CK-ET and CK-ABA interactions exhibit tissue-specific features. CK treatments have been demonstrated to promote the ABA accumulation in shoots, but not in roots, in contrast to ET that accumulates predominantly in roots in response to high CK levels (Žd’árská et al.,, 2013).

## PLANT HORMONES IN RESPONSES TO BIOTIC STRESSES

Hormones also tightly regulate plant responses against pathogens. The networks that control the immune responses in plants are highly complex and have been extensively reviewed (Feys and Parker, 2000; Broekaert et al., 2006; Robert-Seilaniantz et al., 2007; Nishimura and Dangl, 2010). The best characterized hormones that play a role in pathogen response/defense are salicylic acid (SA), jasmonate (JA), and ET. Depending on the lifestyle of the pathogens, a different response will be triggered by the plant. Against biotrophic pathogens, the resistance largely depends on SA-mediated responses and the principal defense strategy is programmed cell death (apoptosis) that restricts the biotrophic pathogen to the infection site, preventing its proliferation, and further spreading in the plant (Dangl and Jones, 2001; Jones and Dangl, 2006; Nishimura and Dangl, 2010; An and Mou, 2011). In contrast, for necrotrophic pathogens that feed on death tissue only, cell death is beneficial. These pathogens induce defense responses that depend on JA and ET to prevent cell death and that trigger the secretion of antimicrobial compounds and the accumulation of proteins with antimicrobial and antifungal activity, such as plant defensins (Overmyer et al., 2000; Andi et al., 2001; Alonso and Stepanova, 2004; Broekaert et al., 2006; Balbi and Devoto, 2008; Fonseca et al., 2009; Gfeller et al., 2010). Because of their difference in the nature of the defense strategy, the JA–ET interaction tends to antagonize the SA responses (Peña-Cortés et al., 1993; Doares et al., 1995; Petersen et al., 2000; Kloek et al., 2001), so that the stress-activated JA–ET signaling might suppress the SA-mediated resistance and vice versa. However, these two pathways might synergistically interact and be considered a fine-tuning mechanism to respond to biotic stresses (Cui et al., 2005; Mur et al., 2006; Truman et al., 2007).

Once the pathogens or microbes have gained access to the plant tissues, they are sensed in each cell by pattern recognition receptors present in the plasma membrane of the host plant cells and bind to microbe-associated molecular patterns (MAMPs; Gómez-Gómez, 2004; Zipfel et al., 2006), the mechanism designated basal resistance or MAMP-triggered immunity (MTI). To overcome MTI, pathogens secrete effectors into the plant cytosol. In this manner, these proteins interfere with the plant immune responses (Chisholm et al., 2006) and modify the host proteins to evade detection and, hence, enhance their virulence, which is referred to as effector-triggered susceptibility. However, the coevolution of plants and microbes has led to the acquisition of the R proteins that specifically recognize these pathogen effectors or avirulence (avr) proteins in a characterized response known as gene-for-gene resistance or effector-triggered immunity (ETI) (Flor, 1971). This specific resistance response is noticeable by localized cell death at the infection site and is known as the hypersensitive response (Hammond-Kosack and Jones, 1996; Greenberg and Yao, 2004).

### SALICYLIC ACID IN BIOTIC STRESSES

During the hypersensitive response, different signal transduction pathways are activated. Tissues distal from the infection site develop an enhanced broad-spectrum resistance to secondary infections that is the systemic acquired resistance (SAR; Yarwood, 1960; Ross, 1961). Before SAR is triggered in remote leaves, SA, which is crucial for this defense strategy, accumulates (Malamy et al., 1990). When transgenic *Arabidopsis* plants express the bacterial SA hydroxylase gene *nahG* that disables the SA accumulation because of its fast turnover to catechol, they cannot develop SAR and induce the pathogen resistance (*PR*) gene expression (Gaffney et al., 1993; Delaney et al., 1994). Furthermore, lipid transfer proteins and SA-binding proteins might be involved in the SA accumulation-triggering signaling in SAR (Park et al., 2007). The non-expresser PR1 (NPR1) protein acts downstream of SA and transduces the signal to promote the *PR* gene expression (Durrant and Dong, 2004). During SAR induction, an oxidative burst occurs, followed by an increase in antioxidants to neutralize the harmful effects of reactive oxygen species. This reducing environment can then convert NPR1 from its inactive oligomeric form into its activated monomeric form that can be transported from the cytosol to the nucleus and activate transcription factors (Kanzaki et al., 2003; Mou et al., 2003), via protein-protein interactions between NPR1 and the TGACG sequence-specific (TGA) transcription factors (Zhang et al., 1999).

### JASMONIC ACID AND ETHYLENE IN BIOTIC STRESSES

The defense response to an attack by necrotrophic pathogens and chewing insects is mediated through the JA pathway that commonly acts together with ET to mount a coordinated defense response. One of the best characterized components of the JA signaling pathway is the coronatine insensitive (COI1) receptor (Devoto et al., 2002; Xu et al., 2002). COI1 is part of the Skp1/Cullin/F-box (SCF) E3 ubiquitin-ligase protein degradation complex SCF^COI1^. High JA levels promote the interaction of the SCF^COI1^ complex with the JA ZIM (JAZ) domain repressors and activate the transcription of JA-responsive genes. The *coi1 *mutants that lack the functional JA receptor are more susceptible to infections by insects and necrotrophic pathogens, such as *Botrytis cinerea*, *Pythium irregulare*, or *Alternaria brassicicola *(van Wees et al., 2003; Adie et al., 2007; Ferrari et al., 2007; Ye et al., 2012). Likewise, mutations that stabilize the JAZ proteins (JAZ1∆3A) increase the susceptibility against herbivores, such as *Spodoptera exigua *(Chung et al., 2008), further supporting the significance of a functional JA signaling pathway in plant defense responses.

The JA-mediated responses against pathogens is strengthened by the ET activity. Ethylene is perceived in plants by the receptors ethylene resistant1 (ETR1), ETR2, ethylene-insensitive4 (EIN4), ethylene response sensor1 (ERS1), and (ERS2) that belong to a histidine kinase family (Bleecker et al., 1988; Chang et al., 1993; Hua et al., 1995, 1998; Sakai et al., 1998). Mutations in these receptors not only confer ET insensitivity, but also increase susceptibility to necrotrophic pathogens (Geraats et al., 2003). Downstream from these receptors, the Raf-like kinase constitutive triple response 1 (CTR1) is active, which is a negative ET response regulator. In the presence of ET, the *CTR1* repression activates EIN2 (Guzmán and Ecker, 1990; Kieber et al., 1993; Chao et al., 1997) and, subsequently, stimulates the EIN3/EIL-like (EIL) transcription factors, whereas mutations in *EIN2* confer ET insensitivity, in addition to an increased susceptibility to necrotrophic pathogens (Geraats et al., 2003).

Although both JA and ET contribute jointly to the plant’s fight against pathogen attacks, the molecular mechanisms of their crosstalk are not well understood, but new insights into the molecular mechanisms underlying their interactions have been provided (Zhu et al., 2011). The JAZ repressors of the JA signaling interact physically with the EIN3/EIL1 transcription factors and attenuate their ability to activate genes (Zhu et al., 2011). This interaction has a striking developmental impact, because it enables JA to contribute to the ET response regulation. Thus, besides the classical mechanism in which ET induces the EIN3/EIL1 stabilization (Guo and Ecker, 2003; Potuschak et al., 2003), EIN3/EIL1 is released from repression by JA through JAZ degradation, thereby triggering ET responses (Zhu et al., 2011).

The hormonal interplay between pathways that depend on JA–ET and SA is particularly important when plants are exposed to multiple pathogens of both biotrophic and necrotrophic types. Under such conditions, an effective defense requires only one of these pathways, but still they need to be tightly balanced with each other. This very complex crosstalk between JA and SA has been reviewed thoroughly (see Beckers and Spoel, 2006; Thaler et al., 2012).

### CYTOKININ AND ITS CROSSTALK WITH SALICYLIC ACID

One of the first indications on the involvement of CK in biotic stress came from tobacco (*Nicotiana tabacum*) plants in which the *S*-adenosyl-homocysteine hydrolases (SAHHs) were downregulated. Originally, SAHHs have been studied in mammals because of their role in the regulation of transmethylation and mRNA 5′ capping during viral replication (De Clercq, 1998). Interestingly, the tobacco plants with low *SAHH* expression not only exhibited an enhanced resistance against the tobacco mosaic virus (TMV), cucumber mosaic virus, potato virus X, and potato virus Y (Masuta et al., 1995), but also increased CK levels and CK-related developmental defects.

In attacked plants, the CK levels are coregulated with the SA levels (Kamada et al., 1992; Sano et al., 1994, 1996; Masuta et al., 1995). Tobacco plants that overexpressed the Ras-related small GTP-binding protein 1 (RGP1)-encoding gene exhibited higher levels of SA and of the acidic pathogenesis-related 1 (*PR-1a*) gene than those of wild-type plants, in correlation with an enhanced resistance against TMV infection. Interestingly, these transgenic plants also showed phenotypes typical for a high endogenous CK activity, such as reduced apical dominance and increased tillering (Kamada et al., 1992), as was, indeed, confirmed later (Sano et al., 1994, 1996). Furthermore, in both wild-type and *RGP1*-overexpressing plants, the CK perception inhibited by the use of the competitive inhibitor 2-chloro-4-cyclohexylamino-6-ethylamino-s-triazine interfered with the expression of the SA-dependent *PR-1a* and the basic JA-dependent *PR-1* after wounding (Sano et al., 1996), thereby suggesting that CK contributes to the defense responses mediated by SA and JA.

**FIGURE 2:**
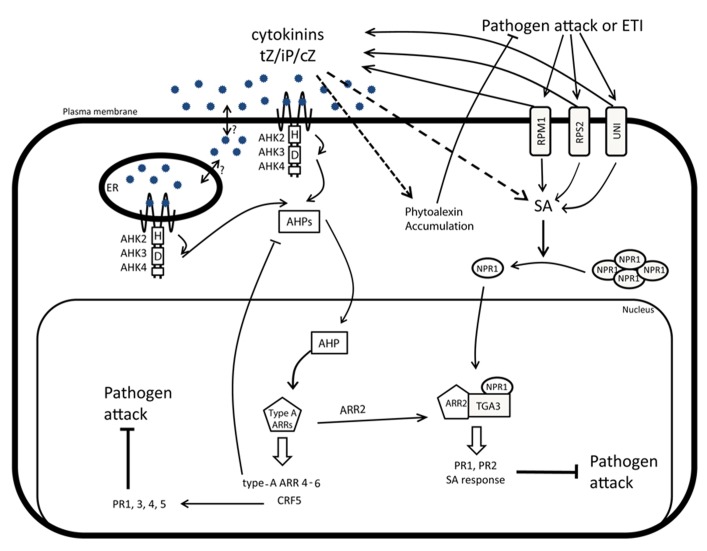
**CK and hormonal crosstalks during biotic stress responses.** Pathogen attacks stimulated by PAMP-triggered immunity (PTI) and effector-triggered immunity (ETI) correlate with a dramatic production of SA and CK. The accumulation of CK will induce the production and accumulation of phytoalexins in a SA-independent manner and also enhance the SA-dependent immunity. In response to pathogens, NPR1 monomerizes and translocates to the nucleus where it interacts with TGA3. The NPR1-TGA3 activity is further regulated through interaction with the type-B ARR2 response regulator, a component of the CK signaling pathway. The TGA3-NPR1-ARR2 complex is required to induce the SA-mediated resistance and to trigger the expression of *PR1* and *PR2*. High CK levels, induced after pathogen attacks, can activate the *CRF5-*mediated branch of the CK signaling pathway and contribute to the regulation of the *PR1*, *PR3*, *PR4*, and *PR5* expression.

As mentioned, the recognition of the pathogen Avr effector proteins by the resistance (R) proteins is an important part in plant defense responses. This interaction triggers ETI, which is characterized by the production of SA and the subsequent induction of *PR* genes and SAR. A dominant-positive mutant of the coiled-coil nucleotide-binding leucine-rich-repeat (CC-NB-LRR) protein UNI (*uni-1D*) that constitutively activates ETI (Igari et al., 2008) exhibits an enhanced expression of *PR-1, PR-5*, and of the type-A ARR CK-signaling repressors and increased endogenous CK levels, with phenotypic alterations typical for high CK activity as a consequence (**Figure 2**; Igari et al., 2008). In *uni-1D *plants, CK levels decreased by the *CKX1* induction reduces both the *PR-1* and of type-A *ARR* gene expression. However, in these *uni-1D *plants, overexpression of the bacterial SA hydroxylase-encoding *nahG* gene prevents SA accumulation and interferes with the *PR-1* expression, but without effect on the type-A *ARR* gene induction and the CK-like phenotypes (Igari et al., 2008). A similar CK-related phenotype has been observed in the knockdown mutant *rin4K-D *of the resistance to *Pseudomonas syringae* pv. *maculicola* (RPM1)-interacting protein 4 (RIN4), which is a negative regulator of R proteins. In *rin4K-D plants, *the R proteins Resistant to *P. syringae* 2 (RPS2) and RPM1 are constitutively active and trigger ETI, whereas both *PR-1* and *ARR5* transcript levels are upregulated and the phenotypic alterations are typical for high CK activity (**Figure 2**; Igari et al., 2008).

Another indication of the crosstalk between CK and SA has emerged from the characterization of the CRF 5 (**Figure 2**; Liang et al., 2010). Indeed, the *CRF5* expression is upregulated in response to *Pseudomonas syringae* pv. *tomato* DC3000 (*Pst* DC3000) and the transcript levels of SA-induced *PR-1*, *PR-3*, *PR-4*, and *PR-5* are increased in the *CRF5*-overexpressing lines (Rashotte et al., 2006; Cutcliffe et al., 2011). This crosstalk mechanism between CK and SA has been elucidated (Choi et al., 2010) by showing that pretreatment of *Arabidopsis *plants with CK significantly increased the resistance against *Pst* DC3000 infection. Correspondingly, mutants defective in CK perception and signaling, such as *ahk2/ahk3* and *arr2, *or plants with reduced endogenous CK levels, such as *35S::CKX2* and *35S::CKX4*, were more susceptible to *Pst* DC3000. In contrast, the plant resistance to Pst DC3000 was enhanced by high endogenous CK levels due to overexpression of the CK biosynthesis (*IPT*) genes or by CK signaling promoted by increased *ARR2* expression (Choi et al., 2010). Therefore, CK has been proposed to affect priming, a defense-related response activation and might assist plants to cope with infections through the induced SA signaling and increased *PR* expression levels (Igari et al., 2008; Choi et al., 2010; Liang et al., 2010). This scenario is strongly supported by the findings that ARR2 interacts directly with the SA response factor TGA3, which binds the promoter regions of *PR-1* and *PR-2*, and that this interaction is essential for the enhanced resistance of the *35S::ARR2* lines. Altogether, both the SA-triggered translocation of NPR1 into the nucleus and the formation of a complex with TGA3-ARR2 are seemingly necessary for the development of a full SA-mediated defense response (Choi et al., 2010, 2011). The impact of CK on the plant defense has been characterized in the *Pst* DC3000-*Arabidopsis* interaction model with the *SA induction deficient 2 (sid2*) mutant that fails to accumulate SA (Naseem et al., 2012). The increased susceptibility of *sid2* toward *Pst* DC3000 can only be partially recovered by CK treatment (Naseem et al., 2012), thereby supporting that CK treatments enhance the immunity in an SA-dependent manner (Naseem and Dandekar, 2012).

Recently, the CK-promoted protection against pathogenic infections has been suggested to be involved in SA-independent mechanisms ((Großkinsky, et al., 2011). In the *P. syringae* pv. *tabaci*-tobacco interaction model, higher CK levels before infection increase the resistance of tobacco against *P. syringae* pv. *tabaci* and this resistance depends on increases phytoalexin levels, such scopoletin and capsidiol, which accumulate in the presence of CK (Großkinsky, et al., 2011).). Thus, the mechanism underlying the CK-mediated resistance of tobacco differs from that in *Arabidopsis* that is based on an SA-dependent transcriptional control. In the solanaceous plant species, CK appears to promote primary defense responses through an increase of the phytoalexin-pathogen ratio in the early infection phases that then efficiently restricts the pathogen development.

### CYTOKININ AND ITS CROSSTALK WITH JASMONIC ACID

Even though there is not much evidence for an interplay between JA and CK, these hormonal pathways might be linked directly (Ueda and Kato, 1982; Dermastia et al., 1994; Sano et al., 1996) and their interaction might be antagonistic (Naik et al., 2002; Stoynova-Bakalova et al., 2008). Typically, in wounded plants, the JA levels increase significantly, whereas the SA levels remain unchanged, but both CK applications and high endogenous CK levels accelerate the defense response to reach a faster maximum release of JA and methyl jasmonate (MeJA) than in control plants (Sano et al., 1996; Dervinis et al., 2010). In potato (*Solanum tuberosum*), JA treatments can induce the accumulation of CK ribosides (Dermastia et al., 1994), whereas they might strongly inhibit the CK-induced callus growth (Ueda and Kato, 1982). These observations hint at a very complex and unexplored interplay, in which the outcome probably depends not only on the CK-JA ratio, but also that of other hormones as well.

### CYTOKININ AND ITS CROSSTALK WITH AUXIN

Crosstalk between CK and auxin has been widely studied over the years, particularly in a developmental context in which their interaction is primarily antagonistic (Bishopp et al., 2011; Vanstraelen and Benková, 2012), although a number of recent studies undoubtedly point toward a role of auxin in stress responses. Various pathogens can produce auxins or modulate auxin levels *in planta* to enhance the plant susceptibility to infection (Chen et al., 2007; An and Mou, 2011). In *Arabidopsis* plants lacking the functional *RPS2* gene, the expression of the *P. syringae* type III effector *AvrRpt2* decreased the resistance against *Pst* DC3000, and also show altered auxin levels and auxin-related phenotypes (Chen et al., 2007). This direct correlation between sensitivity and auxin levels implies that auxin promotes plant susceptibility. Also, a recent study in which *PR1* was used as a marker gene in the *Pst* DC3000–*Arabidopsis* interaction revealed that, whereas the immunity was positively promoted by CK and SA, it was negatively regulated by auxin, JA, and ABA (Naseem et al., 2012). Interestingly, the positive effect of CK pretreatments on the plant immunity can be repressed by a combined CK and auxin treatment (Naseem et al., 2012). Based on this evidence, CK and auxin might play a highly possible antagonistic role in plant defense responses, but the specific mechanisms that modulate this crosstalk are still unknown.

A model for the CK–auxin interplay in plant defense has been proposed (Naseem and Dandekar, 2012). After infection, pathogens will modulate the auxin levels and the signaling that will diminish the responses mediated by SA and CK, whereas CK pretreatments will prevent the auxin-based susceptibility, due to the known effect of CK on auxin transport and signaling.

## CONCLUSIONS AND FUTURE PERSPECTIVES

Nowadays, one of the major objectives of plant biologists is to improve plant performances under less favorable environmental conditions. By enhancing plant defense responses against biotic and abiotic stress, non-cultivable land might be used, the losses due flooding and infections be decreased, and the amount of applied fertilizers and pesticides in the fields be reduced. However, because the crosstalk between stress-related and developmental hormones is largely unknown, and uncharacterized, usually unforeseen problems occur when the stress resistance is modified. Ideally, plants with enhance resistance to stress or pathogen attacks should not be affected in growth or developmentally hampered. In this context, it is crucial to understand the hormonal crosstalks underlying plant responses to various stresses, because the modification of one single hormonal pathway will very probably alter the activity of other hormonal pathways as well.

The complexity of the impact of hormones on the resistance to stress can be nicely illustrated with examples of plants with altered CK levels. Due to the importance of CK in stress responses, several genes involved in the regulation of CK levels have been proposed as possible targets to enhance stress resistance, such as the *IPT* and *CKX* genes (Werner et al., 2010; Nishiyama et al., 2011; Wang et al., 2011b). However, the benefit of the stress-tolerant phenotype of the *IPT *loss-of-function**mutants or of *CKX*-overexpressing plants was counteracted by developmental defects caused by low bioactive CK levels, such as N6-(∆2-isopentenyl)adenine and *trans*-zeatin (Nishiyama et al., 2011). To overcome this drawback, it is necessary to control the CK activity either in an organ or in a tissue-specific manner, an approach that has already been used in several species (McCabe et al., 2001; S’ýkorová et al., 2008; Ghanem et al., 2011; Qin et al., 2011). For instance, as a consequence of downregulated CK levels in root tissues only (Werner et al., 2010), root length, branching, and biomass increased and the plants were also more resistant to abiotic stress treatments, such as severe drought or heavy metal contaminations (Werner et al., 2010). Furthermore, modulation of CK-mediated defense to stress might at the same time attenuate the input provided by other signaling pathways, such as ABA (Wang et al., 2011b). A reduced CK content leads to a decrease in ABA content and hypersensitivity to ABA treatments (Nishiyama et al., 2011), in contrast to the stressed plants in which the ABA levels are upregulated (Stoll et al., 2000; Hansen and Dörffling, 2003; Kudoyarova et al., 2007). Correspondingly, overexpresssion of *IPT8 *results in insensitivity to ABA treatments and prevents the induction of *ABI1* and *ABI5* in seedlings (Wang et al., 2011b). These examples clearly show that a good knowledge of the molecular mechanisms underlying the hormone-mediated responses and of the mutual communication among hormonal pathways might be very rewarding in the targeted modulation of specific hormonal pathways and, hence, in the effective plant adaptation to concrete environmental conditions.

Extended studies on the genes that mediate the crosstalk between CK and other developmental and stress-related hormones might identify novel targets for the stress tolerance improvement of crop species. Importantly, the identification of molecular components and mechanisms that mediate the phytohormonal interplay might enable us to dissect the stress-related from the developmental functions.

Finally, to increase the plant resistance against various stresses, new alternative approaches should take in account the specific features of the plant species and the distinct mechanisms that underlay their stress responses (Choi et al., 2010; Großkinskyet al., 2011). A nice example of such a strategy is the enhanced drought stress tolerance of alfalfa (*Medicago sativa*) by means of CK-overproducing *Sinorhizobium meliloti* without impact on nitrogen fixation (Xu et al., 2012).

## Conflict of Interest Statement

The authors declare that the research was conducted in the absence of any commercial or financial relationships that could be construed as a potential conflict of interest.
